# Dietary Nitrate Supplementation and Exercise Performance

**DOI:** 10.1007/s40279-014-0149-y

**Published:** 2014-05-03

**Authors:** Andrew M. Jones

**Affiliations:** Sport and Health Sciences, College of Life and Environmental Sciences, University of Exeter, St. Luke’s Campus, Exeter, EX1 2LU UK

## Abstract

Dietary nitrate is growing in popularity as a sports nutrition supplement. This article reviews the evidence base for the potential of inorganic nitrate to enhance sports and exercise performance. Inorganic nitrate is present in numerous foodstuffs and is abundant in green leafy vegetables and beetroot. Following ingestion, nitrate is converted in the body to nitrite and stored and circulated in the blood. In conditions of low oxygen availability, nitrite can be converted into nitric oxide, which is known to play a number of important roles in vascular and metabolic control. Dietary nitrate supplementation increases plasma nitrite concentration and reduces resting blood pressure. Intriguingly, nitrate supplementation also reduces the oxygen cost of submaximal exercise and can, in some circumstances, enhance exercise tolerance and performance. The mechanisms that may be responsible for these effects are reviewed and practical guidelines for safe and efficacious dietary nitrate supplementation are provided.

## Introduction

**Fig. 1:**
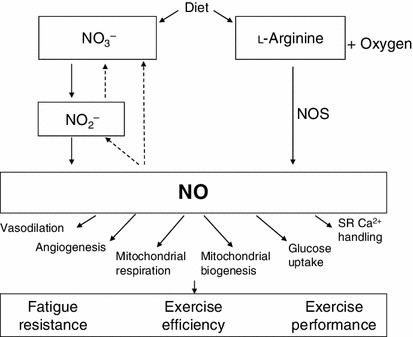
Pathways of NO production. NO is produced from the substrates l-arginine and oxygen in a reaction catalyzed by the NO synthases and is subsequently oxidized to nitrite and nitrate. Nitrate can be reduced to nitrite by xanthine oxidase and by anaerobic bacteria in the oral cavity, and nitrite can be further reduced to NO and other reactive nitrogen species, an effect that is accentuated when tissue oxygen availability is low. In this way, the products of NO production can be recycled. In addition to endogenous production, body stores of nitrate and nitrite can be increased through the consumption of foods that are rich in inorganic nitrate such as green leafy vegetables and some fruits. NO is important in several physiological processes that may support or enhance exercise performance. It is possible that the reliance on the nitrate–nitrite–NO pathway for NO production is increased during exercise. *Dashed arrows* show that NO can be oxidized to NO_2_^−^ and NO_3_^−^. *Ca* ^*2+*^ calcium, *NO* nitric oxide, *NO*_*3*_^−^ nitrate, *NO*_*2*_^−^ nitrite, *NOS* nitric oxide synthase, *SR* sarcoplasmic reticulum

Until relatively recently, it was believed that the ubiquitous physiological signaling molecule, nitric oxide (NO), was generated solely through the oxidation of l-arginine in a reaction catalyzed by a family of NO synthase (NOS) enzymes, resulting in the endogenous production of nitrate (NO_3_^−^) and nitrite (NO_2_^−^) [1] (Fig. 1). However, it is now known that nitrate and nitrite can be reduced back to NO and other bioactive nitrogen oxides in vivo, and there is growing scientific interest in the potential of this ‘nitrate–nitrite–NO’ pathway in physiology, dietetics, and medicine [2–5]. It has been suggested that this alternative pathway may complement the l-arginine–NOS–NO pathway by enabling NO production in conditions of low oxygen availability in which NOS activity (which is oxygen dependent) may be reduced [6].

In addition to its generation by means of the NOS system, body stores of nitrate and nitrite may also be increased exogenously through the diet, particularly through the consumption of green leafy vegetables such as lettuce, spinach, rocket, celery, cress, and beetroot, which typically contain over 250 mg (>4 mmol) nitrate per 100 g fresh weight [2, 5, 7]. Ingested inorganic nitrate circulates in the plasma, and a portion (~25 %) is taken up by the salivary glands and concentrated in the saliva [8]. Commensal facultative anaerobic bacteria residing in the crypts on the surface of the tongue reduce nitrate to nitrite [9, 10]. Some of the swallowed nitrite is reduced to NO in the acidic environment of the stomach, but a substantial amount of nitrite enters the systemic circulation, elevating the plasma nitrite concentration ([nitrite]) [11, 12]. Following bolus nitrate ingestion, plasma nitrate concentration ([nitrate]) peaks after 1–2 h and plasma [nitrite] peaks after 2–3 h, after which both gradually fall, arriving back at baseline values after about 24 h [13]. A variety of enzymes and proteins, including deoxyhemoglobin, can subsequently catalyze the one-electron reduction of nitrite to NO in blood and other tissues [14–16]. This process is facilitated in conditions of low oxygen availability (ischemia and hypoxia) and low pH [17], enabling NO to be produced where it is most required. Interestingly, these conditions (low partial pressure oxygen and pH) may exist in skeletal muscle during exercise [18].

There is reason to believe that enhancing NO bioavailability by augmenting the nitrate–nitrite–NO pathway may influence muscle function and exercise performance. NO can modulate skeletal muscle function through its role in the regulation of blood flow, contractility, glucose and calcium homeostasis, and mitochondrial respiration and biogenesis [19]. In vivo, NOS inhibition, which would reduce endogenous NO production, increases oxygen consumption ($\dot{V}{\sc{\text {O}}}_2$) in dogs [20] and rats [21] and also influences $\dot{V}{\sc{\text {O}}}_2$ dynamics in horses [22]. In humans, the influence of NOS blockade is more controversial, but there are indications that NO is involved in the regulation of blood flow and $\dot{V}{\sc{\text {O}}}_2$ [23–25]. This raises the possibility that augmenting NO bioavailability might positively influence exercise performance. Indeed, several studies have reported that plasma [nitrite] is positively associated with exercise capacity in humans [26–29]. Collectively, these findings raise the possibility that augmenting plasma [nitrite] through dietary nitrate supplementation might have ergogenic effects.

The purpose of this article is to provide an overview of the accumulating research evidence that points to a role for enhancing NO bioavailability, by dietary nitrate supplementation, in the physiological responses to exercise and exercise performance. Due to space restrictions, the focus of the article is entirely on the influence of nitrate on the responses of young, healthy, physically active individuals to exercise in normoxia. It is recognized that nitrate may also be beneficial for cardiovascular health in the general population due to reductions in blood pressure [13, 14, 30], during exercise in hypoxia [31, 32], and also in older and some clinical populations [33, 34].

## Nitrate Reduces Oxygen Cost of Exercise and Improves Exercise Tolerance

The first study to show that nitrate supplementation may improve exercise efficiency was published in 2007 by Larsen et al. [35]. In that study, nine well trained subjects consumed 0.1 mmol/kg body mass (BM) per day of sodium nitrate or a placebo for 3 days before completing a continuous incremental cycle ergometer test. Nitrate supplementation significantly elevated resting plasma [nitrite] (by 82 %) and reduced resting systolic and diastolic blood pressure (by 8 and 6 mmHg). The oxygen cost of exercise over the first four stages of the test was significantly reduced after nitrate supplementation compared with placebo, with a mean reduction in $\dot{V}{\sc{\text {O}}}_2$ of 5 %, but there was no significant difference in $\dot{V}{\sc{\text{O}}}_{2{\text {max}}}$ between treatments. Over the four lowest work rates, there were significant improvements in gross efficiency (calculated as the work output per unit energy expended, from 19.7 ± 1.6 to 21.1 ± 1.3 %) and delta efficiency (calculated as the change in work output per unit change in energy expended, from 22.1 ± 1.6 to 22.9 ± 1.9 %) following nitrate supplementation. There was no difference in blood [lactate], heart rate, ventilation, or respiratory exchange ratio between nitrate and placebo for any of the submaximal work rates (corresponding to 45–80 % $\dot{V}{\sc{\text{O}}}_{2{\text {max}}}$). Although indirect, these results indicate no change in non-oxidative energy supply, the energy cost of cardiopulmonary processes, or substrate utilization following nitrate supplementation, and suggest instead a real effect on the efficiency of muscle oxidative metabolism.

The findings of Larsen et al. [35] are remarkable because a basic tenet of human exercise physiology is that the oxygen cost of submaximal exercise at a given work rate is immutable, that is, essentially fixed, irrespective of age, health, and fitness status, and insensitive to known physical, nutritional, or pharmacological interventions [36]. It is well established that endurance exercise performance is a function of $\dot{V}{\sc{\text{O}}}_{2{\text {max}}}$, the fractional utilization of $\dot{V}{\sc{\text{O}}}_{2{\text {max}}}$, and exercise efficiency [37, 38]. Assuming that the other factors remain unchanged, an improvement in muscle efficiency would be expected to enable a greater work output for the same energy cost and translate into improved exercise performance [37, 38].

Recognizing the potential importance of the results of Larsen et al. [35] to the enhancement of performance, Bailey et al. [39] examined the influence of dietary nitrate supplementation on $\dot{V}{\sc{\text {O}}}_2$ dynamics during step exercise tests of moderate intensity (80 % of the gas exchange threshold [GET]) and high intensity (70 % of the difference between the GET and $\dot{V}{\sc{\text{O}}}_{2{\text {max}}}$), with the latter tests continued to exhaustion as a measure of exercise tolerance. An important difference from the study of Larsen et al. [35] was that Bailey et al. [39] used a natural nitrate-rich dietary source, beetroot juice, as the nitrate supplement. Eight healthy men consumed 0.5 l/day of either beetroot juice (5.6 mmol nitrate) or blackcurrant cordial as a placebo for 6 consecutive days, with the exercise tests completed on the last 3 days. On days 4–6 of the supplementation periods, plasma [nitrite] was significantly elevated following nitrate intake compared with placebo (95 %), and systolic blood pressure was significantly reduced (by an average of 8 mmHg). During moderate-intensity exercise, steady-state $\dot{V}{\sc{\text {O}}}_2$ was reduced by 5 %. During high-intensity exercise, there was a significant 23 % reduction in the amplitude of the $\dot{V}{\sc{\text {O}}}_2$ ‘slow component,’ which is considered to reflect a progressive loss of muscle efficiency as high-intensity exercise proceeds [40]. The peak $\dot{V}{\sc{\text {O}}}_2$ attained during high-intensity exercise was not different between treatments, but attainment of the peak $\dot{V}{\sc{\text {O}}}_2$ was delayed with nitrate such that the time to exhaustion was significantly increased (by 16 %; from 9.7 ± 2.4 to 11.3 ± 3.4 min). Consistent with the study of Larsen et al. [35], nitrate did not alter blood [lactate], heart rate, ventilation, or respiratory exchange ratio for either moderate- or high-intensity exercise. In a subsequent study using knee extensor exercise, Bailey et al. [41] confirmed that, compared with placebo, beetroot juice consumption reduced submaximal $\dot{V}{\sc{\text {O}}}_2$ during low-intensity exercise, reduced the $\dot{V}{\sc{\text {O}}}_2$ slow component, and increased time to exhaustion by 25 % (from 9.8 ± 1.3 to 12.2 ± 1.8 min) during high-intensity exercise.

In a follow-up study, Larsen et al. [42] reported that dietary supplementation with sodium nitrate (0.1 mmol/kg BM/day) for 2 days significantly reduced $\dot{V}{\sc{\text{O}}}_{2{\text {max}}}$ (from 3.72 ± 0.33 to 3.62 ± 0.31 l/min, i.e. 2.7 %) during a maximal incremental exercise test involving combined arm and leg cranking. Despite this small reduction in $\dot{V}{\sc{\text{O}}}_{2{\text {max}}}$, the time to exhaustion during the incremental test was 7 % longer after nitrate supplementation than with placebo (8.7 ± 0.5–9.4 ± 0.5 min, *p* = 0.13). The authors concluded that dietary nitrate reduces $\dot{V}{\sc{\text{O}}}_{2{\text {max}}}$ during maximal exercise using a large active muscle mass but with a trend towards improved performance due to enhanced muscle energetic function. This finding of a lower $\dot{V}{\sc{\text{O}}}_{2{\text {max}}}$ following nitrate supplementation, either with no change or with improved exercise performance, was also found by Bescós et al. [43], but is by no means a universal finding.

**Fig. 2:**
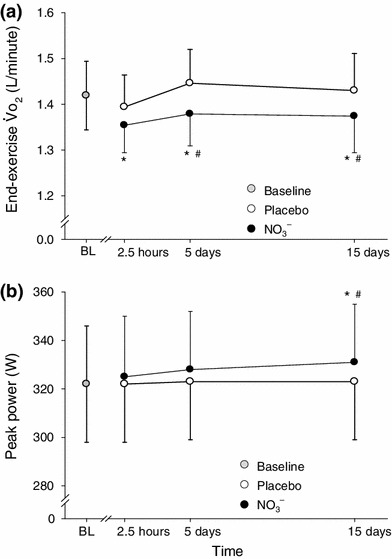
Influence of acute and chronic dietary nitrate supplementation with 0.5 l/day of beetroot juice on **a** pulmonary oxygen uptake during submaximal exercise and **b** peak power output achieved during ramp incremental exercise. The nitrate condition is shown in *filled symbols*, the placebo condition is shown in *open symbols*, and the non-supplemented control condition is shown in *grey symbols*. The steady-state $\dot{V}{\sc{\text{O}}}_2$ during moderate-intensity exercise was reduced 2.5 h after nitrate ingestion and this was maintained after 5 and 15 days of continued supplementation. The peak power output was higher than the other conditions after 15 days of supplementation. Values are mean ± SD. *Significantly different from the non-supplemented control condition (*p* *<* 0.05); ^*#*^significantly different from the placebo condition (*p* *<* 0.05). *BL* baseline, $\dot{V}{\sc {O_2}}$ oxygen uptake

The duration of the supplementation period in the early studies into the effects of nitrate supplementation on exercise efficiency and performance was typically 3 days followed by a maintenance dose over the remaining 1–3 days of experimentation [35, 39, 41, 42]. It was therefore not known whether shorter or longer periods of nitrate supplementation might be more, or less, effective in altering exercise efficiency and performance. Vanhatalo et al. [44] addressed this issue by asking eight healthy volunteers to consume 0.5 l/day of beetroot juice (5.2 mmol nitrate/day) or a placebo for 15 days. Another key difference compared with previous studies was that subjects were asked not to alter their normal diet during the supplementation period, that is, they were free to continue consuming nitrate-rich foodstuffs. The exercise protocol (two moderate-intensity step tests followed by a maximal ramp incremental test on a cycle ergometer) was completed acutely (2.5 h after beetroot juice or placebo ingestion) and after 5 and 15 days of supplementation. The steady-state $\dot{V}{\sc{\text{O}}}_2$ during moderate-intensity exercise was significantly reduced (by approximately 4 %) 2.5 h after beetroot juice intake compared with pre-supplementation control, and it remained significantly reduced after 5 and 15 days of supplementation compared with placebo (Fig. 2). The results also showed that the effect of nitrate on efficiency is maintained (neither lost nor enhanced) for at least 2 weeks if supplementation is continued. Finally, the results indicate that the effects on efficiency are still manifest, albeit perhaps less impressively, when normal dietary nitrate intake is not restricted. Interestingly, there was no improvement in ramp test performance, relative to placebo, after 2.5 h or 5 days of beetroot juice intake. However, there were significant increases in the peak power output and the power output at the GET compared with placebo, after 15 days of beetroot juice supplementation (Fig. 2).

In addition to nitrate, beetroot juice also contains other compounds that may be independently bioactive or that may act synergistically with nitrate, including antioxidants (betaine and vitamins) and the polyphenols, resveratrol and quercetin [45]. An important scientific development was therefore the creation of a nitrate-depleted beetroot juice placebo that was used first in the study of Lansley et al. [46]. In that study, nine healthy subjects consumed 0.5 l of beetroot juice/day (6.2 mmol of nitrate) or the same volume of placebo (<0.005 mmol of nitrate) for 6 days and completed treadmill and knee-extension exercise tests on days 4–6. The oxygen cost of treadmill walking and moderate-intensity treadmill running were significantly reduced. During high-intensity running, beetroot juice supplementation resulted in an enhanced exercise tolerance of 15 % (7.5 ± 1.7, 7.6 ± 1.5, and 8.7 ± 1.8 min for pre-supplemented control, placebo, and beetroot juice conditions, respectively). The time to exhaustion during incremental knee-extension exercise was also significantly longer following nitrate supplementation (8.2 ± 0.9, 8.2 ± 0.9, and 8.5 ± 0.8 min for pre-supplemented control, placebo, and beetroot juice conditions, respectively). The lack of effect of nitrate-depleted beetroot juice consumption on plasma [nitrite], blood pressure, the oxygen cost of exercise, and exercise tolerance in this study point clearly to the nitrate content, per se, being chiefly responsible for the physiological effects of beetroot juice consumption.

## Effects on Exercise Performance

It is important to recognize that, while valuable scientifically, the time to exhaustion or incremental tests used in the early nitrate supplementation studies [39, 41, 42, 44, 46] are tests of exercise capacity rather than exercise performance. ‘Real-world’ competitive sport typically requires that athletes complete a given distance in the shortest possible time. It is also important to note that the effect size in time to exhaustion tests is considerably greater than in time trials. For example, a 15 % improvement in time to exhaustion following a given intervention might be expected to translate into a 1 % improvement in time trial performance over an equivalent duration [47]. Although seemingly small, such an effect would be highly meaningful in performance terms to an elite athlete.

Recognizing the importance of evaluating the influence of dietary nitrate supplementation on exercise performance, Lansley et al. [48] asked nine club-level male cyclists (mean $\dot{V}{\sc{\text{O}}}_{2{\text {max}}}$, 56 ml/kg per minute) to complete both a 4-km and a 16.1-km time trial on a cycle ergometer following the acute consumption of 0.5 l of beetroot juice (6.2 mmol of nitrate) or nitrate-depleted beetroot juice as a placebo. Because acute nitrate ingestion had previously been shown to reduce steady-state $\dot{V}{\sc{\text{O}}}_2$ [44], the beverages were consumed 2.5 h before the commencement of the time trials. Given that nitrate supplementation reduces $\dot{V}{\sc{\text{O}}}_2$ for the same fixed power output [35, 39, 44], the authors hypothesized that, during a time trial, the cyclists would be able to produce a higher power output for the same $\dot{V}{\sc{\text{O}}}_2$ and thus complete the distance in less time. Consistent with the experimental hypothesis, $\dot{V}{\sc{\text{O}}}_2$ during the time trials was not significantly different between the treatments, but beetroot juice significantly increased the mean power output during the 4- and 16.1-km time trials. Consequently, compared with placebo, beetroot juice significantly improved 4-km performance (2.8 %) and 16.1-km performance (2.7 %).

Cermak et al. [49] also examined the influence of nitrate supplementation on cycle time trial performance. These authors tested the effect of 6 days of concentrated beetroot juice ingestion (8 mmol nitrate/day) on 10-km cycle time trial performance in 12 trained male cyclists (mean $\dot{V}{\sc{\text{O}}}_{2{\text {max}}}$, 58 mL/kg per minute). After supplementation on the 6th day, the subjects completed 60 min of submaximal cycling (2 × 30 min at 45 and 65 % of peak power output), followed by a 10-km time trial. Time trial performance (15.9 ± 0.3 vs. 16.1 ± 0.3 min) and power output (294 ± 12 vs. 288 ± 12 W) were significantly improved after beetroot juice compared with placebo supplementation. Submaximal $\dot{V}{\sc{\text{O}}}_2$ was significantly lower (5 %) after beetroot juice than after placebo at both 45 and 65 % of peak power output. Whole-body fuel selection and plasma lactate, glucose, and insulin concentrations did not differ between treatments. Collectively, these studies suggest that both acute [48] and more chronic [49] dietary nitrate supplementation improves cycle efficiency and time trial performance, at least for events of approximately 5–30 min duration in trained but sub-elite cyclists ($\dot{V}{\sc{\text{O}}}_{2{\text {max}}}$ of approximately 53–63 ml/kg per minute).

Two other studies have reported positive effects of nitrate supplementation on exercise performance. Murphy et al. [50] reported that ‘recreationally fit’ adults completed a 5-km treadmill time trial faster (*p* = 0.06) following the consumption of 200 g baked beetroot (≥500 mg or ≥8 mmol nitrate) 75 min before exercise compared with consumption of cranberry relish as an eucaloric placebo. At 1.8 km into the 5-km run, the rating of perceived exertion was significantly lower and running speed over the last 1.8 km of the 5-km distance was 5 % faster in the beetroot trial. Bond et al. [51] tested the effect of 6 days of 0.5 l of beetroot juice or placebo consumption on six 500-m rowing ergometer performances in 14 well trained rowers. The authors concluded that beetroot juice supplementation conferred a likely benefit to mean repetition time compared with placebo (0.4 %, 95 % confidence limits ±1.0 %) and an almost certain benefit for repetitions 4–6 (1.7 %, 95 % confidence limits ±1.0 %).

**Fig. 3:**
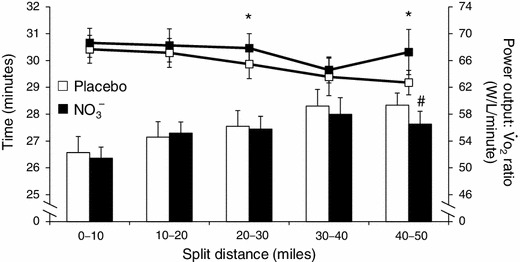
Influence of dietary nitrate (*black symbols*) and placebo (*white symbols*) supplementation on 50-mile cycling time trial performance. The *lines* in the *upper* part of the figure show the ratio of power output to $\dot{V}{\sc{\text{O}}}_2$ and the *bars* in the *lower* part of the figure show the split times taken to complete consecutive 10-mile splits. The power output to $\dot{V}{\sc{\text{O}}}_2$ ratio was higher for the 20- to 30-mile (*p = 0.05) and 40- 50-mile (*p < 0.05) splits, and the 40- to 50-mile split was completed in a faster time (^#^ *p* < 0.05) following nitrate supplementation compared with placebo. However, there was no significant difference in overall performance between treatments (beetroot juice 136.7 ± 5.6 vs. placebo 137.9 ± 6.4 min). Values are shown as mean ± SD. $\dot{V}{\sc {O_2}}$ oxygen uptake

More recently, several studies have reported no ergogenic effect of acute [52–54] or short-term [55] nitrate supplementation on exercise performance in highly trained endurance athletes ($\dot{V}{\sc{\text{O}}}_{2{\text {max}}}$, 60–70 ml/kg per minute). Wilkerson et al. [54] asked eight well trained cyclists to complete a 50-mile time trial 2.5 h following the consumption of 0.5 l of beetroot juice or placebo. There was no significant difference in time trial performance between treatments (beetroot juice 136.7 ± 5.6 vs. placebo 137.9 ± 6.4 min), although there was a tendency for the ratio of power output to $\dot{V}{\sc{\text{O}}}_2$ to be higher following nitrate supplementation (Fig. 3). An interesting feature of the study was the relatively small mean increase in plasma [nitrite] following nitrate ingestion compared with less well trained subjects and the suggestion of ‘responders’ and ‘non–responders’ to the treatment. There was a significant inverse correlation between the increased post-beverage plasma [nitrite] and the reduction in time trial completion time (*r* = −0.83, *p* = 0.01). The authors concluded that the high training status of the cyclists studied and/or the long duration and relatively low intensity of the 50-mile time trial might have reduced the potential for nitrate supplementation to improve performance.

Consistent with these results, Cermak et al. [52] reported that trained cyclists (mean $\dot{V}{\sc{\text{O}}}_{2{\text {max}}}$, 60 ml/kg per minute, *n* = 20) did not cover more distance in a 1 h cycling time trial following the acute ingestion of concentrated beetroot juice (8.7 mmol nitrate) compared with placebo. Similarly, Peacock et al. [53] found that the consumption of 614 mg (~10 mmol) of potassium nitrate 2.5 h before exercise neither reduced submaximal $\dot{V}{\sc{\text{O}}}_2$ during a low-intensity warm-up nor enhanced 5-km running performance compared with placebo in ten highly trained cross-country skiers ($\dot{V}{\sc{\text{O}}}_{2{\text {max}}}$, 70 ml/kg per minute). Using a 3-day nitrate supplementation protocol (10 mg/kg or 0.16 mmol/kg BM sodium nitrate/day), Bescós et al. [55] found no difference compared with placebo on 40-min cycle time trial performance in trained cyclists and triathletes (mean $\dot{V}{\sc{\text{O}}}_{2{\text {max}}}$, 60 ml/kg per minute).

As noted by Wilkerson et al. [54], there are several reasons why both the training/aerobic fitness status of the subjects and the intensity of the exercise task might influence whether nitrate supplementation is ergogenic. Highly trained subjects are likely to have high NOS activity [56], which might render the nitrate–nitrite–NO pathway relatively less important for NO production. Moreover, highly trained subjects may have higher plasma [nitrite] values than sedentary or lesser trained subjects [57], such that the response to a standard dose of nitrate may be diminished. It should also be recalled that nitrite is reduced to NO in hypoxic (and acidic) conditions, in which NOS function is simultaneously compromised. Highly trained subjects would be expected to have greater skeletal muscle capillarization [58], perhaps minimizing any hypoperfusion of metabolically active tissue during exercise, and therefore reducing the requirement for NO production through the reduction of nitrite. For the same reason, low-intensity endurance exercise, in which skeletal muscle remains well oxygenated and pH does not fall significantly, would presumably not result in obligatory NO production from nitrite. Finally, recent evidence that nitrate supplementation may preferentially alter contractile function in type II fibers [59] would suggest that endurance athletes, who typically evince a low proportion of such fibers in the trained musculature [60], might experience a blunted physiological response to nitrate supplementation.

To date, the studies showing negligible effects of nitrate in elite athletes have used acute (2–3 h pre-performance [52–54]) or short-term (3 day [55]) supplementation protocols. In contrast, mechanistic studies (see Sect. 4), which have indicated that nitrate alters muscle contractile [59] and mitochondrial [61] proteins, employed longer (3–7 day) supplementation periods. This raises the possibility that longer-term supplementation and/or higher nitrate doses may be required to improve performance in elite athletes. Indeed, the results of Cermak et al. [49] and Vanhatalo et al. [44] suggest the possibility that longer-term supplementation may be more effective than acute supplementation for enhancing performance. There are anecdotal reports that beetroot juice supplementation was used extensively, and successfully, by members of several prominent national teams competing in a wide variety of sports at the 2012 London Olympic and Paralympic Games. Additional research is required into the ergogenic potential of nitrate supplementation in elite athletes.

## Mechanisms

The mechanistic bases for the lower oxygen cost of exercise, implying improved muscle efficiency, and the enhanced exercise tolerance following dietary nitrate supplementation have been addressed in several recent studies [41, 59, 61]. Given the ubiquity and multifarious roles of NO in physiology, it is not surprising that there are several possible molecular explanations for the reported systemic effects of nitrate supplementation.

Bailey et al. [41] used ^31^P-magnetic resonance spectroscopy to examine changes in skeletal muscle energetics following nitrate supplementation. Over the final 3 days of a 6-day supplementation period during which subjects consumed 0.5 l beetroot juice (5.1 mmol nitrate) or placebo/day, the subjects completed low- and high-intensity knee extensor exercise tests using a custom-built ergometer both in the exercise physiology laboratory (for measurement of pulmonary gas exchange) and within the bore of a superconducting magnet. During both low- and high-intensity exercise, pulmonary $\dot{V}{\sc{\text{O}}}_2$ was significantly reduced following nitrate supplementation, consistent with earlier reports [35, 39]. This reduction in $\dot{V}{\sc{\text{O}}}_2$ was associated with a sparing of intramuscular phosphocreatine concentration (PCr) and a blunting of the increases in adenosine diphosphate concentration (ADP) and inorganic phosphate (P_i_) concentration. There was no difference in muscle pH between treatments at any time, indicating that there was no ‘compensatory’ increased contribution of anaerobic glycolysis to energy turnover as may be the case if, for example, nitrate supplementation had resulted in an inhibition of respiration [62, 63].

The results of Bailey et al. [41] were important in demonstrating that the reduction in the whole-body oxygen cost of exercise following nitrate supplementation is consequent to changes in muscle energy metabolism. The proportional sparing of $\dot{V}{\sc{\text{O}}}_2$ and PCr reported by Bailey et al. [41] indicated that nitrate supplementation may alter the energy (adenosine triphosphate [ATP]) cost of muscle power production. The authors suggested that this could occur by the possible effects of NO on the sarcoplasmic reticulum calcium (Ca^2+^) ATPase or the actin-myosin ATPase [64–66]. A lower ATP cost of force production would blunt the changes in intramuscular substrates and metabolites that stimulate mitochondrial respiration (e.g. PCr, ADP, P_i_) [67, 68], and could explain the lower oxygen cost of exercise. Interestingly, the depletion of muscle PCr and the accumulation of P_i_ and ADP have been linked with the process of muscle fatigue during high-intensity exercise [69]. The blunted changes in energy substrates and metabolites might therefore help to explain the improved exercise tolerance observed following nitrate supplementation. An alternative explanation for the coincident reductions in steady-state $\dot{V}{\sc{\text{O}}}_2$ and PCr is that nitrate supplementation simultaneously improves muscle oxygenation (thus sparing muscle PCr) [70] and improves mitochondrial efficiency (thus lowering $\dot{V}{\sc{\text{O}}}_2$).

Evidence for positive effects of nitrate supplementation on both mitochondrial efficiency [61] and muscle contractile function [59] has recently been presented. Larsen et al. [61] asked 14 healthy volunteers to consume 0.1 mmol/kg BM/day of sodium nitrate or a placebo for 3 days, after which a muscle biopsy was taken and a submaximal exercise test was completed. Following nitrate supplementation, the expression of adenine nucleotide translocase (ANT), a protein involved in mitochondrial proton conductance, was reduced, thus reducing leak respiration and improving the efficiency of oxidative phosphorylation. Nitrate supplementation resulted in a 19 % increase in the mitochondrial P/O ratio (the amount of oxygen consumed per ATP produced), which was closely correlated (*r* = −0.80) with the reduction in whole-body $\dot{V}{\sc{\text{O}}}_2$ during submaximal cycling. These results indicate that the reduced $\dot{V}{\sc{\text{O}}}_2$ during exercise following nitrate supplementation is related to a reduced leakage/slippage of protons across the inner mitochondrial membrane. The authors speculated that nitrate supplementation might result in an increased inhibition of cytochrome c oxidase by NO [62, 63], which might be sensed by the cell as mild hypoxia, initiating signaling mechanisms that result in a downregulation of ANT and improved mitochondrial efficiency. Interestingly, in contrast to 3 days of in vivo nitrate administration, the acute application of nitrite to isolated mitochondria in vitro had no acute effect on the P/O ratio. This finding suggests that several days of nitrate treatment may be required for the induction of changes in the expression of relevant mitochondrial proteins such as ANT.

However, the effects of nitrate supplementation on muscle function may not be confined to the mitochondria. Hernández et al. [59] have reported improvements in muscle Ca^2+^ handling and contractile function in mice fed sodium nitrate in water for 7 days compared with age-matched controls that received water without added nitrate. In particular, in fast-twitch muscle fibers, nitrate supplementation increased myoplasmic free Ca^2+^ concentration at stimulation frequencies from 20 to 150 Hz, effects that were related to the increased expression of calsequestrin 1 and the dihydropyridine receptor, proteins that are involved in Ca^2+^ handling. These striking effects on intracellular Ca^2+^ handling resulted in significantly increased contractile force at 50 Hz or less and a faster rate of force development at 100 Hz stimulation. There were no effects of nitrate supplementation on muscle proteins or contractile force in slow-twitch muscles. The authors concluded that dietary nitrate intake in humans may increase muscle function during normal movement. The results of Hernández et al. [59] are consistent with the suggestion of Bailey et al. [41] that the effects of nitrate on muscle efficiency may be explained, at least in part, by extramitochondrial mechanisms.

In addition to evoking these intracellular effects, there is recent evidence that nitrate supplementation might also enhance blood flow to contracting muscle. Ferguson et al. [71] fed beetroot juice (or a water placebo) to rats for 5 days, and then measured blood pressure and hind limb muscle blood flow during submaximal treadmill running. Exercising blood pressure and blood [lactate] were significantly lower following nitrate feeding, and there was a striking (38 %) increase in muscle blood flow between nitrate-fed and placebo-fed rats. The greater muscle blood flow was directed preferentially towards hind limb muscles expressing a high fraction of type II muscle fibers. Muscle oxygen delivery was therefore substantially elevated in the low oxidative, highly fatigable fibers, such that oxygen might be considered to be more appropriately distributed across and within the active muscles. This might be expected to reduce substrate-level phosphorylation, improve metabolic control and exercise efficiency, and be advantageous to performance [40]. In a subsequent study, the same authors reported that microvascular oxygen pressure fell less rapidly following the onset of electrically evoked contractions of the spinotrapezius muscle of rats fed beetroot juice compared with those fed water [72]. This is consistent with a greater oxygen driving pressure across the transition from rest to exercise. Those studies [71, 72] indicate that nitrate supplementation, which may reduce both the ATP and oxygen cost of muscle contraction [41, 59, 61], simultaneously increases muscle oxygen delivery. The net result is a higher ratio of oxygen delivery to oxygen utilization, which would be expected to reduce the muscle metabolic perturbation and be conducive to muscle fatigue resistance.

## Practical Considerations

From the above review, it appears clear that dietary nitrate has the potential to reduce blood pressure, lower the oxygen cost of exercise, and, at least in some circumstances, enhance exercise capacity. However, very little is known about the nitrate intake that may optimize these positive effects while minimizing any potential risks to health. For example, while it is known that supplementation with approximately 5–9 mmol of nitrate/day for 1–15 days can elicit favorable effects on the physiological responses to exercise, the dose–response relationship has yet to be established. It should be emphasized that 5–9 mmol nitrate can readily be consumed within the normal diet and there is presently no evidence that additional nitrate intake produces greater benefits.

With regard to the effect of nitrate on indices of exercise performance in healthy volunteers, the literature appears consistent in showing that 2–6 days (or up to 15 days) of supplementation can increase indices of performance during high-intensity constant work-rate exercise and maximal incremental exercise [39, 41, 44, 46]. The effects of acute supplementation on performance are less consistent, with some studies showing a positive effect [33, 48, 50, 73] and others showing no effect [52–54, 74]. It is likely that the efficacy of acute nitrate supplementation will depend on several factors such as the age, health, diet, and fitness/training status (including muscle fiber type proportions, capillarization, and baseline plasma [nitrite]) of the subjects tested; the intensity, duration, and nature of the exercise task; and whether the exercise is performed in normoxia or hypoxia. Acute nitrate intake may rapidly influence vascular tone and peripheral tissue oxygenation [33, 44], but more time may be necessary to permit changes in mitochondrial and contractile proteins to influence exercise performance [59, 61]. Whether longer-term nitrate supplementation may support or augment (or even hinder) the physiological adaptations to training is presently unknown.

The duration of continuous maximal exercise for which nitrate appears to be ergogenic is in the range of 5–30 min [39, 41, 46, 48–51]. There is limited evidence that nitrate is beneficial for longer duration exercise (>40 min) performance, at least when administered acutely [52, 54, 55]. This may be related to the lower intensity of such exercise and the associated reduced likelihood of the development of local mismatching of perfusion to metabolic rate in muscle (i.e. loci that are relatively hypoxic and acidic). Whether nitrate supplementation may be ergogenic during very high-intensity continuous or intermittent exercise has not been systematically evaluated. However, two studies indicate that high-intensity intermittent exercise performance might be enhanced by nitrate supplementation [51, 73].

Nitrite and nitrate occur naturally in vegetables and are also added to cured and processed meats to ‘fix’ color and delay spoilage. The potential for the formation of nitrosamines in food has stimulated debate about the safety of ingested nitrite [75, 76]. There are only limited data on the effects of dietary nitrate supplementation by athletes on the production of nitrosamines. Larsen et al. [61] reported no difference in tyrosine nitration of skeletal muscle proteins between sodium nitrate (3 days at 0.1 mmol/kg BM/day) and placebo treatments, and Bescós et al. [55] found no difference in urinary nitrated protein levels between sodium nitrate (3 days at 10 mg/kg or 0.16 mmol/kg BM nitrate) and placebo treatments. It is pertinent to note that any possible harmful effect of nitrosation (which assumes the presence of secondary amines in the saliva or stomach) is very effectively inhibited [77] by the antioxidants that accompany nitrate in vegetables [45]. However, although nitrate itself is not toxic due to its limited and slow conversion to nitrite, there is the possibility of toxicity with the accidental or uncontrolled use of nitrite salts [78]. The expert consensus view is that nitrate supplementation with vegetable products such as beetroot juice is very unlikely to be harmful [78, 79]. Athletes wishing to explore the possible ergogenic properties of nitrate supplementation are therefore advised to use natural vegetable products for this purpose. Although unlikely, it is not known whether longer-term intake of high-nitrate vegetable products may be health damaging, and further longer-term studies are required before chronic supplementation can be recommended.

## Conclusions

In summary, dietary nitrate supplementation appears to represent a promising new approach for enhancing aspects of the physiological response to exercise, such as muscle efficiency and oxygenation, which might augment performance. However, research is at an early stage and the precise conditions in which nitrate may be ergogenic have yet to be firmly established. For example, the efficacy of nitrate might well depend on factors such as the type of subject, including age, diet, and health and fitness status; the intensity, duration, and nature of the exercise challenge; and the dose applied and duration of the nitrate supplementation regimen. Time will tell.
